# Potential of wind power projects under the Clean Development Mechanism in India

**DOI:** 10.1186/1750-0680-2-8

**Published:** 2007-07-30

**Authors:** Pallav Purohit, Axel Michaelowa

**Affiliations:** 1 Research Programme on International Climate Policy, Hamburg Institute of International Economics (HWWI), Neuer Jungfernstieg 21, D-20347 Hamburg, Germany; 2 Political Economy and Development, Institute of Political Science, University of Zurich, Mühlegasse 21, 8001 Zurich, Switzerland

## Abstract

**Background:**

So far, the cumulative installed capacity of wind power projects in India is far below their gross potential (≤ 15%) despite very high level of policy support, tax benefits, long term financing schemes etc., for more than 10 years etc. One of the major barriers is the high costs of investments in these systems. The Clean Development Mechanism (CDM) of the Kyoto Protocol provides industrialized countries with an incentive to invest in emission reduction projects in developing countries to achieve a reduction in CO_2 _emissions at lowest cost that also promotes sustainable development in the host country. Wind power projects could be of interest under the CDM because they directly displace greenhouse gas emissions while contributing to sustainable rural development, if developed correctly.

**Results:**

Our estimates indicate that there is a vast theoretical potential of CO_2 _mitigation by the use of wind energy in India. The annual potential Certified Emissions Reductions (CERs) of wind power projects in India could theoretically reach 86 million. Under more realistic assumptions about diffusion of wind power projects based on past experiences with the government-run programmes, annual CER volumes by 2012 could reach 41 to 67 million and 78 to 83 million by 2020.

**Conclusion:**

The projections based on the past diffusion trend indicate that in India, even with highly favorable assumptions, the dissemination of wind power projects is not likely to reach its maximum estimated potential in another 15 years. CDM could help to achieve the maximum utilization potential more rapidly as compared to the current diffusion trend if supportive policies are introduced.

## Background: Indian Energy Demand, Wind Energy Technology, Technical Potential of Wind Energy and its Support in India

The global energy demand is expected to grow at a staggering rate in the next 30 years. The International Energy Agency [1] predicts that the world's energy needs will be almost 60% higher in 2030 than they are now. Two-thirds of this increase will arise in China, India and other rapidly developing economies, which will account for almost half the energy consumption by 2030. Sharp increases in world energy demand will trigger important investments in generating capacity and grid infrastructure. According to the IEA, the global power sector will need to build some 4,800 GW of new capacity between now and 2030.

**Figure 1:**
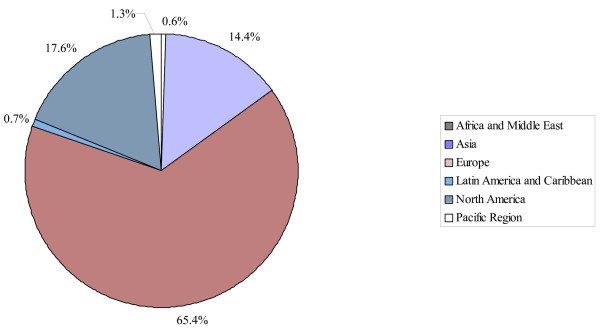
Regional distribution of the global installed wind power capacity (Source: [8]).

In the 11^th ^Five Year Plan, the Government of India aims to achieve a GDP growth rate of 10% and maintain an average growth of about 8% in the next 15 years [2]. According to Indian government officials, the growth of Indian economy is highly dependent on the growth on its energy consumption [3]. The 2006 capacity of power plants in India was 124 GW, of which 66% thermal, 25% hydro, 3% nuclear and 5% new renewables [4]. At the same time, Chinese power capacity reached over 600 GW [5], showing India's backlog. Wind energy is an alternative clean energy source and has been the world's fastest growing renewable energy source growing at a rate of 28% in the last decade [6]. Wind power has the advantage of being harnessed on a local basis for application in rural and remote areas [7]. Global wind power capacity reached 74 GW at the end of 2006 [8], 13 countries had more than 1 GW installed. Figure 1 presents the regional distribution of the global installed wind power capacity [8].

The impetus behind wind power expansion has come increasingly from the urgent need to combat global climate change. Most countries now accept that greenhouse gas (GHG) emissions must be drastically slashed in order to avoid environmental catastrophe. Wind energy offers both a power source that completely avoids the emission of carbon dioxide, the main GHG, but also produces none of the other pollutants associated with either fossil fuel or nuclear generation [9]. Wind power can deliver industrial scale on-grid capacity. Starting from the 1997 Kyoto Protocol, a series of GHG reduction targets has cascaded down to a regional and national level. These in turn have been translated into targets for increasing the proportion of renewable energy, including wind. In order to achieve these targets, countries in both Europe and elsewhere have adopted a variety of market support mechanisms [10-15]. These range from premium payments per unit of output to more complex mechanisms based on an obligation on power suppliers to source a rising percentage of their supply from renewables. As the market has grown, wind power has shown a dramatic fall in cost [16]. The production cost of a kilowatt-hour of wind power is one fifth of what it was 20 years ago. In the best locations, wind is already competitive with new coal-fired plants. Individual wind turbines have also increased in capacity, with the standard commercial machines reaching 2.5 MW and prototypes for offshore plants even 5 MW.

The successful wind energy business has attracted the serious attention of the banking and investment market, with new players such as oil companies entering the market. Hays and Attwood [17] concludes that Asia is playing an increasingly important role in the global wind industry as the region prepares to invest over $12 billion in wind power generation capacity in the second half of this decade. In India, wind power already occupies a prominent position with regard to installed capacity – reaching 6.2 GW by the end of 2006. In 2006 alone, an aggregate capacity of 1.8 GW has been added [8]. Thus, India is the fourth largest wind market in the world [18]. However, the total installed capacity of wind power projects still remains far below from their respective potential (i. e. <15%). One of the barriers to the large-scale dissemination of wind power projects in India is the high upfront cost of these systems [19]. Other barriers to wind power projects are low plant load factors, unstable policies of the state governments and poor institutional framework.

**Figure 2:**
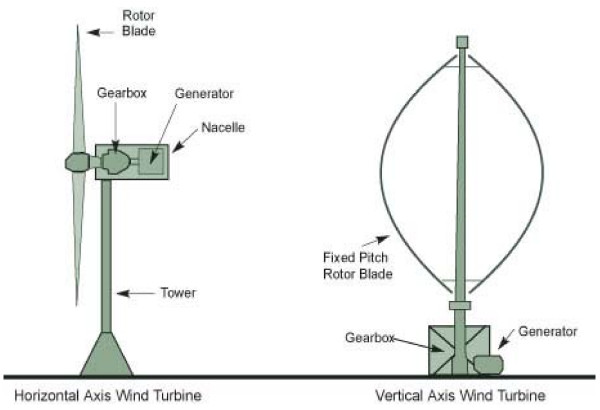
Schematic of the horizontal and vertical axis wind turbine (Source: [23]).

Wind has considerable amount of kinetic energy when blowing at high speeds [20]. This kinetic energy when passing through the blades of the wind turbines is converted into mechanical energy and rotates the wind blades [21] and the connected generator, thereby producing electricity. A wind turbine primarily consists of a main tower, blades, nacelle, hub, main shaft, gearbox, bearing and housing, brake, and generator [22]. The main tower is 50–100 m high. Generally, three blades made up of Fiber Reinforced Polyester are mounted on the hub, while in the nacelle the major parts are housed. Under normal operating conditions the nacelle would be facing the upstream wind direction [20]. The hub connects the gearbox and the blades. Solid high carbon steel bars or cylinders are used as main shaft. The gearbox is used to increase the speed ratio so that the rotor speed is increased to the rated generator speed [21]; it is the most critical component and needs regular maintenance. Oil cooling is employed to control the heating of the gearbox. Gearboxes are mounted over dampers to minimize vibration. Failure of gearbox may put the plant out of operation for an entire season as spares are often not available. Thus, new gearless configurations have become attractive for wind plant operators. Modern turbines fall into two basic groups: horizontal axis turbines and vertical axis turbines as shown in Figure 2[23]. Horizontal axis turbines resemble airplane propellers, with two to three rotor blades fixed at the front of the tower and facing into the wind. This is the most common design found today, making up most of the large utility-scale turbines on the global market. Vertical axis turbines resemble a large eggbeater with rotor blades attached vertically at the top and near the bottom of the tower and bulging out in the middle.

The most dramatic improvement has been in the increasing size and performance of wind turbines. From machines of just 25 kW twenty years ago, the commercial size range sold today is typically from 600 up to 2,500 kW, with 80 m diameter rotors placed on 70–100 m high towers. In 2003, the German company Enercon erected the first prototype of a 4.5 MW turbine with a rotor diameter of 112 m. Wind turbines have a design lifetime of 20–25 years, with their operation and maintenance costs typically about 3–5% of the cost of the turbine. For the share of different wind turbine types in India see Table 1.

**Table 1:** Manufacturers-wise wind electric generators installed in India (as on 31.03.2006)

**S. No.**	**Manufacturer**	**Rating (kW)**	**Details as on 31^st ^March 2006**	
			**Numbers**	**Capacity (in MW)**

1	ABAN – Kenetech	410	231	94.71
2	AMTL – Wind World	220	2	0.44
		250	328	82.00
		500	3	1.50
3	BHEL	55	16	0.88
		200	17	3.40
4	BHEL Nordex	200	79	15.80
		250	184	46.00
5	C-WEL	250	57	14.25
		600	2	1.20
6	Danish Windpower	150	12	1.80
7	Das Lagerwey	80	9	0.72
		250	284	71.00
8	Elecon	200	1	0.20
		300	51	15.30
		600	5	3.0
9	Enercon	230	451	103.73
		330	38	12.54
		600	681	408.60
		800	435	348
10	GE Wind Energy	1500	12	18
11	Himalaya	140	4	0.56
		200	24	4.80
12	JMP-Ecotecnia	225	10	2.25
13	Kirloskar – WEG	400	8	3.20
14	Micon (Pearl)	90	99	8.91
15	Mitsubishi	315	6	1.89
16	Nedwind-Windia	250	4	1.00
		500	20	10.00
		550	35	19.25
17	NEG Micon	750	674	505.5
		950	54	51.30
		1650	137	226.05
18	NEPC India	225	957	215.325
		250	16	4.0
		400	7	2.80
		750	12	9.0
19	NEPC-Micon	55	14	0.77
		110	2	0.22
		200	50	10.00
		225	589	132.53
		250	528	132.00
		400	121	48.40
		600	2	1.20
20	Pegasus	250	9	2.25
21	Pioneer Asia	850	35	29.75
22	Pioneer Wincon	110	10	1.10
		250	260	65.00
		755	1	0.76
23	REPL-Bonus	55	22	1.21
		100	1	0.10
		320	60	19.20
24	RES-Adavanced Wind Turbine	250	80	20.00
25	Sangeeth – Carter	300	25	7.50
26	Suzlon	270	2	0.54
		350	836	292.60
		600	15	9.0
		1000	81	81.00
		1250	1255	1568.75
		2000	1	2.00
27	Tacke	250	4	1.00
		600	21	12.60
		750	1	0.75
28	Textool-Nordtank	300	65	19.50
		550	5	2.75
29	TTG/Shriram EPC	250	230	57.50
30	Vestas – RRB	55	31	1.71
		90	21	1.89
		100	5	0.50
		200	56	11.20
		225	735	165.375
		500	562	281.00
		600	65	
31	Wind Master	200	1	0.20
32	Windmatic	55	30	1.65
33	Wind Power	330	29	9.57

	**TOTAL**		**10825**	**5340.96**

Source: [24]

At present, efforts are being made to develop a low cost, indigenous, horizontal axis Wind Energy Generator (WEG) of 500 kW rating. The WEG will have a two bladed rotor and the tower will be a tubular tower with guys. The organizations contributing in the development of the WEG are (i) National Aerospace Laboratory (NAL), (ii) Structural Engineering Research Centre (SERC), (iii) Sangeet Group of Companies, and (iv) Center for Wind Energy Technology (C-WET). It will be specially suited for Indian wind conditions i.e. relatively low wind speeds and dusty environment. It is further learnt that this WEG may cost almost 50% as compared to the other WEGs of the same rating commercially available in India. The WEG is nearing completion and likely to be completed by April-2007 [24].

Wind in India is dominated by the strong south-west summer monsoon, which starts in May–June, when cool, humid air moves towards the land and the weaker north-east winter monsoon, which starts in October, when cool, dry air moves towards the ocean. During the period March to August, the wind is uniformly strong over the whole Indian Peninsula, except the eastern peninsular coast. Wind speeds during the period November to March are relatively weak, though higher winds are available during a part of the period on the Tamil Nadu coastline.

**Figure 3:**
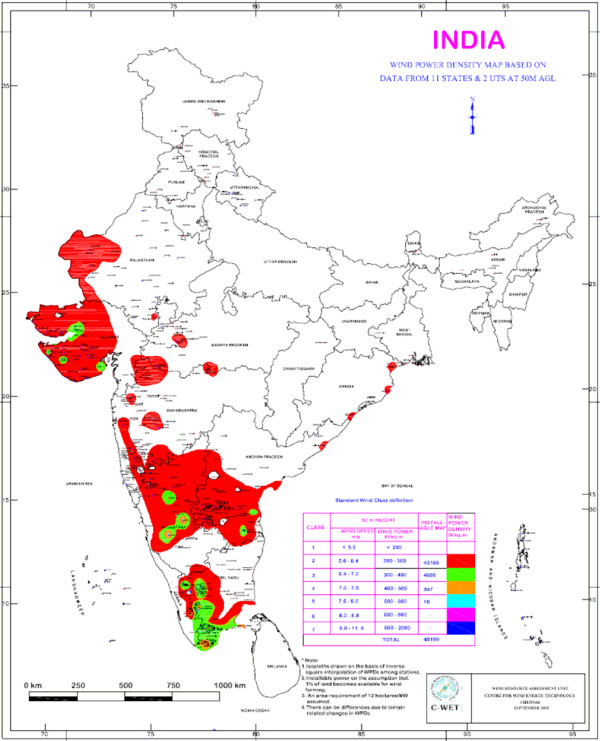
Wind power potential in India (Source: Centre for Wind Energy Technology (C-WET), Government of India).

In order to tap the potential of wind energy sources, there is a need to assess the availability of the resources spatially. A Wind Resource Assessment Programme was taken up in India in 1985 [19]. Around 1150 wind monitoring/mapping stations were set up in 25 states and Union Territories (UTs) for this purpose. Over 200 wind monitoring stations in 13 states and UTs with annual mean wind power density greater than 200 W/m^2 ^at a height of 50 m above the ground level show wind speeds suitable for wind power generation [25]. The wind power density at a height of 50 m above the ground level is depicted in Figure 3.

On a regional basis, more detailed assessments have been done. Ramachandra and Shruthi [26] employed a geographical information system (GIS) to map the wind energy resources of Karnataka state and analyzed their variability considering spatial and seasonal aspects. A spatial data base with data of wind velocities has been developed and used for evaluation of the theoretical potential through continuous monitoring and mapping of the wind resources. The study shows that the average wind velocity in Karnataka varies from 0.9 m/s in Bagalkote to 8.3 m/s in Chikkodi during the monsoon season. Agroclimatic zone wise analysis shows that the northern dry zone and the central dry zone are ideally suited for harvesting wind energy for regional economic development.

Onshore wind power potential in the country has been assessed at 45 GW assuming 1% of land availability for wind power generation in the potential areas [27]. However, it is estimated that a penetration (supply fraction) of wind power on a large grid can be as much as 15–20% without affecting grid stability due to requirement of reactive power [28]. Therefore, at present, it is not technically feasible to exploit the full wind power potential in view of total installed power-generating capacities from conventional power generating methods including hydro-electric power plants in different states. Considering a maximum of 20% penetration of existing capacities of the grids through wind power in the potential states, technical potential for grid interactive wind power is presently limited to only 13 GW [25]. Total technical potential for wind power in the country is expected to increase with augmentation of grid capacity in potential states. Table 2 presents a state wise break-up of the estimated technical potential along with wind power installed capacity as on 30 September 2006. One should note that Tamil Nadu has already surpassed the presumed technical potential, indicating that it may be underestimated for India as a whole.

**Table 2:** State wise gross wind power potential, technical potential and cumulative installed capacity in India up to 30.09.2006

**State**	**Gross potential (MW)**	**Technical potential (MW)**	**Installed capacity (MW)**
Andhra Pradesh	8275	1750	121
Gujarat	9675	1780	376
Karnataka	6620	1120	688
Kerala	875	605	2
Madhya Pradesh	5500	825	53
Maharashtra	3650	3020	1242
Orissa	1700	680	2
Rajasthan	5400	895	386
Tamil Nadu	3050	1750	3148
West Bengal	450	450	2

**Total (All India)**	**45195**	**12875**	**6018**

Source: [25]

The original impetus to develop wind energy in India came in the early 1980s from the government, when the Commission for Additional Sources of Energy had been set up in 1981 and upgraded to the Department of Non-Conventional Energy Sources in 1982. The setup of these institutions was due to the wish to encourage a diversification of fuel sources away from the growing demand for coal, oil and gas required to meet the demand of the country's rapid economic growth [29]. A market-oriented strategy was adopted from inception, which has led to the successful commercial development of the technology. The broad based national programme included wind resource assessment; research and development support; implementation of demonstration projects to create awareness and opening up of new sites; involvement of utilities and industry; development of infrastructure capability and capacity for manufacture, installation, operation and maintenance of wind power plants; and policy support.

**Figure 4:**
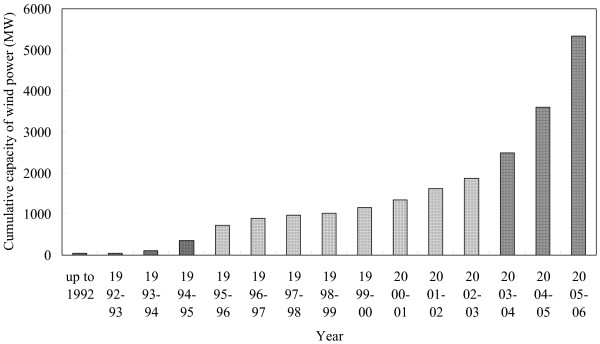
Development of wind power capacity in India over time (Source: MNES Annual Reports).

The Ministry of Non-Conventional Energy Sources (MNES) which was set up in 1992 has been providing support for research and development, survey and assessment of wind resources, demonstration of wind energy technologies and has also taken fiscal and promotional measures for implementation of private sector projects [30,31]. India now has a fairly well-developed and growing wind power industry with a number of Indian companies involved in manufacturing of wind turbines. These companies have tied up with foreign wind power industries for joint venture/licensed production in India, for their market shares see Table 1. Wind turbines up to 2 MW are presently manufactured in India [25]. Figure 4 presents the cumulative capacity of wind power installed in India over time.

A notable feature of the Indian programme has been the interest among private investors/developers in setting up of commercial wind power projects. This is due to a range of fiscal incentives provided by the Indian government such as 80% accelerated depreciation, tax holiday for power generation projects, concessional customs and excise duty as well as liberalized foreign investment procedures [25,29,31]. The Indian Renewable Energy Development Agency (IREDA) provides concessional loans. Current interest rates are 9.5% for a maximum repayment period of 10 years and 9.0% for a maximum repayment period of 8 years [25]. Table 3 presents the summary of key central government incentives for wind power projects in India.

**Table 3:** Financial and fiscal incentives for wind power projects in India

**Type of incentive**	**Description**	**Rate**
**I. Indirect Taxes**	i) Wind operated electricity generators upto 30 kW and wind operated battery chargers upto 30 kW.	5%
	ii) Parts of wind operated electricity generators for manufacturer of wind operated electricity generators, namely: Special bearing, Gear Box, Yaw components, Wind turbine controllers.	5%
	Sensors, Brake hydraulics, Flexible coupling, Brake callipers.	25%
	iii) Blades for rotor of wind operated electricity generators for the manufacturers or the manufacturers of wind operated electricity generators.	5%
	iv) Parts for the manufacturer or the maintenance of blades for rotor of wind operated electricity generation.	5%
	v) Raw materials for manufacturer of blades for rotor of wind operated electricity generators.	5%

**II. Excise Duty**	Devices/Systems exempted from Excise Duty:	
	i) Wind operated electricity generator, its components and parts thereof including rotor and wind turbine controller.	
	ii) Water pumping wind mills, wind aero-generators and battery chargers. [Notification No.6/2002 dated 01/03/2002 (S.No.237 non-conventional devices/systems)]	

**III. Sales Tax**	Exemption/reduction in Central Sales Tax and General Sales Tax are available on sale of renewable energy equipment in various states.	
	i) 80% Accelerated Depreciation on specified Non-conventional Renewable Energy devices/systems (including wind power equipment) in the first year of installation of the projects.	
	ii) Tax Holiday on Power Projects.	

Sources: Ministry of Non-conventional Energy Sources, Government of India, New Delhi

The MNES has issued guidelines to all state governments to create an attractive environment for the export, purchase, wheeling and banking of electricity generated by wind power projects. The guidelines include the promotion of renewables including wind energy through preferential tariffs and a minimum obligation on distribution companies to source a certain share of electricity from renewable energy. However, only a subset of states is actually complying with these guidelines. The State Electricity Regulatory Commissions (SERCs)of Andhra Pradesh, Madhya Pradesh, Karnataka and Maharashtra provide preferential tariffs for wind power. Maharashtra, Andhra Pradesh, Karnataka, Madhya Pradesh and Orissahave enacted the renewables obligation on distributors. The problem with incentives on the state level is that they vary erratically and thus cannot be taken for granted by project developers (see Table 4 for the case of Rajasthan).

**Table 4:** Policy of the state of Rajasthan for sale of power from wind installations

**Period**	**Policy of the state of Rajasthan for sale of power from wind installations**
March 1999 – February 2000	Electricity could be purchased at INR 2.75 (US$ 0.061/kWh) with just 2% wheeling charges along with sales tax incentives. The developer was allowed to bank electricity for one year.
February 2000 – April 2003	Electricity could be purchased at INR 3.03 (US$ 0.067/kWh) while the wheeling charges were kept same at 2%. The provision for banking for 12 months was limited to end of financial year only (March 31). If the banking period is exhausted and the electricity was not sold out by then, the state power utility would buy balance amount of electricity at 60% of the agreed purchase price.
April 2003 – October 2004	Electricity could be purchased at INR 3.32 (US$ 0.073/kWh). The wheeling charges were drastically increased from 2% to 10% for the volume of electricity supplied to the grid. The banking period was reduced from 12 months to the end of calendar year (December 31).

October 2004 – Onwards	The purchase price was reduced from INR 3.32/kWh (US$ 0.073/kWh) to INR 2.91/kWh (US$ 0.064/kWh) which is 13% lower than the previous power policy.

Source: [33]

The main attraction for private investment is the fact that owning a wind turbine assures a profitable power supply compared to the industrial power tariff, which is kept artificially high to cross-subsidize electricity tariffs for farmers. Therefore, clusters of individually owned wind turbines appear to substitute grid electricity. More than 97% of investment in the Indian wind sector is provided from the private sector [25]. However, the impending liberalization under the Electricity Act 2003 may take away this key incentive if industrial power users can procure electricity at competitive rates.

The Clean Development Mechanism under the Kyoto Protocol allows developing countries to generate emission credits (CERs) for industrialized countries by GHG emission reduction projects such as wind power. The sale of CERs could help to accelerate wind power development in India. We assess the theoretical CDM potential of wind power projects in India before discussing whether at the current market situation such projects could become attractive.

## Results: CDM Potential of Wind Power Projects in India

Considerable variation has been observed in the reported values of the PLF of the wind power plants in the CDM Project Design Documents (Table 5). Therefore, in this study to estimate the CDM potential of wind power projects in India the PLF of the wind power plants have been taken as 25%. There are five regional grids within the country – the Northern, Western, Southern, Eastern and North-Eastern. Therefore, the CO_2 _emissions mitigation potential through wind power projects in India is estimated on the basis of the regional grids, whose emission factors have been calculated by the Central Electricity Authority (CEA) of the Government of India in 2006. Table 6 presents the estimated values of CDM potential through wind power projects in India on the basis of the regional baselines.

**Table 5:** Additionality Test of Indian projects on wind power

**Title**	**Methodology**	**Investment Analysis**	**Barrier Analysis**	**Investment and Barrier Analysis**	**Identification of alternatives**	**Institutional/Regulatory Barriers**	**Technology Barriers**	**Common Practice Analysis**	**Impact of CDM registration**	**Remarks**
Nagda Hills (6.25 MW) Wind Energy Project	AMS-I.D.	√	√, I/IR/PP	√	√	√	×	√	√	PLF = 29%; IRR without CDM ~ 9.8%; IRR with CDM ~ 13.5%.
12.3 MW wind energy project in Tamil nadu, India	AMS-I.D.	√	√, I/IR/T/PP	√	×	√	√	√	√	PLF = 22%; IRR without CDM ~ 12.9%; IRR with CDM ~ 13.4%.
14.8 MW small-scale grid connected wind power project in Jaisalmer state Rajasthan	AMS-I.D.	√	√, I/PP	√	×	×	×	√	×	The PLF was considered as 25% before the WEGs started operating, it was later found out to be less than 18%. IRR of the project activity reduced to less than 10% after the execution of the project.
Bundled Wind power project in Jaisalmer, Rajasthan (58.2 MW)	ACM2	√	√, I/IR/PP	√	√	×	×	√	√	PLF at 22.28% (IRR = 9.2% without CDM and 14.6% with CDM); PLF at 25.28% (IRR = 11.0% without CDM and IRR = 17.1% with CDM).
Bundled wind power project in Chitradurga (Karnataka in India) managed by Enercon (India) Ltd. (16.8 MW)	ACM2	√	√, I/PP	√	√	×	×	√	√	PLF at 26% (IRR = 9.5% without CDM and 11.5% with CDM); PLF at 30% (IRR = 14.8% without CDM and IRR = 17.4% with CDM).
3.75 MW Small Scale Grid Connected "Demonstration Wind Farm Project" at Chalkewadi, District Satara, State Maharashtra	AMS-I.D.	×	√, I/IR/T	×	×	√	√	×	×	PLF = 18 – 20%; The investor saw CDM revenue as a risk mitigation against these uncertainties.
11.35 MW Grid Connected Wind Electricity Project at Pohra (Rajasthan)	AMS-I.D.	×	√, I/IR/T	×	×	√	√	×	×	PLF = 20 – 22%; The investor saw CDM revenue as a risk mitigation against these uncertainties.
10.6 MW wind farm at Village Badabagh, District Jaisalmer, Rajasthan.	AMS-I.D.	×	√, I/IR/T	×	×	√	√	×	×	PLF varies from 14.7 to 22.5%.
56.25 MW bundled wind energy project in Tirunelveli and Coimbatore districts in Tamilnadu	ACM2	√	√, I/PP	√	√	×	×	√	√	PLF = 14 – 17.5%; IRR = 10.1% without CDM and IRR = 12.1% with CDM.
5 MW Wind Project at Baramsar and Soda Mada, Jaisalmer, Rajasthan	AMS-I.D.	×	√, I/IR/T	×	×	√	√	×	×	Investment barriers exists.
7.5 MW wind farm of REI Agro Ltd. at Soda-Mada in the state of Rajasthan	AMS-I.D.	×	√, I/IR	×	×	√	×	×	×	Investment barriers exists.
11.25 MW wind power project in Dhule, Maharashtra, India	AMS-I.D.	√	√, I	√	×	×	×	×	×	IRR without CDM ~ 14.17%; IRR with CDM ~ 21.59% which is above the acceptable bench mark IRR of 15.06%.
Wind Electricity Generation at Erakandurai, Dist:Tirunavalli by M/s GHCL Ltd	AMS-I.D.	√	√, I	√	×	×	×	×	×	PLF at 22.83%; IRR = 11.54% without CDM and 14.70% with CDM. Similarly, with PLF at 21.43%; IRR = 9.72% without CDM and 12.58% with CDM.
125 MW wind power project in Karnataka	ACM2	√	√, I/PP	√	√	√	√	√	√	IRR = 7.36% without CDM revenues and 7.87% with CDM revenues.
Generation of electricity from 6.25 MW capacity wind mills by Sun-n-Sand Hotels Pvt. Ltd at Soda Mada Rajasthan	AMS-I.D.	√	√, I/IR/T/PP	√	√	√	√	√	√	PLF = 17 – 19%; IRR without CDM ~ 12.45%; IRR with CDM ~ 14.81%
Generation of electricity from 4 MW capacity wind mills by Sun-n-Sand Hotel group at Supa, Maharashtra	AMS-I.D.	√	√, I/IR/T/PP	√	√	√	√	√	√	PLF = 20%; IRR without CDM ~ 13.76%; IRR with CDM ~ 16.53%
Generation of electricity from 2.5 MW capacity wind mills by Gujarat JHM Hotels Ltd. Ltd at Soda Mada, Rajasthan	AMS-I.D.	√	√, I/IR/T/PP	√	√	√	√	√	√	PLF = 17 – 19%; IRR without CDM ~ 10.57%; IRR with CDM ~ 12.93%
Generation of electricity from 1.2 MW capacity wind mills by Sun-n-Sand Hotels Pvt. Ltd at Satara, Maharashtra	AMS-I.D.	√	√, I/IR/T/PP	√	√	√	√	√	√	PLF = 22 – 25%; IRR without CDM ~ 16.84%; IRR with CDM ~ 19.86%.
15.4 MW wind farm at Satara District, Maharashtra*	ACM2	×	√, I/IR	×	√	√	×	×	√	PLF = 19.24%
4.2 MW Wind power project in Maharashtra, by Bharat Forge Limited*	AMS-I.D.	√	√, I/IR/T	√	×	√	√	×	√	PLF = 13.09 – 23.96%; IRR without CDM ~ 14.3%; IRR with CDM ~ 16.4%.

I: Investment barrier; T: Technological barrier; I/R.: Institutional and/or regulatory barriers; PP: Barriers due to the prevailing practice. *Reg. request

Source: [33]

**Table 6:** Annual gross and technical CO_2 _emissions mitigation potential through wind power projects in India

State	Region	Baseline* (kg CO_2_/kWh)	Annual electricity generation (TWh)		Annual CO_2 _emissions mitigation potential (million tonnes)	
			
			Gross	Technical	Gross	Technical
Andhra Pradesh	Southern	0.86	18.1	3.8	15.6	3.3
Gujarat	Western	0.89	21.2	3.9	19.0	3.5
Karnataka	Southern	0.86	14.5	2.5	12.5	2.1
Kerala	Southern	0.86	1.9	1.3	1.6	1.1
Madhya Pradesh	Western	0.89	12.0	1.8	10.8	1.6
Maharashtra	Western	0.89	8.0	6.6	7.2	5.9
Orissa	Eastern	1.04	3.7	1.5	3.9	1.5
Rajasthan	Northern	0.75	11.8	2.0	8.9	1.5
Tamilnadu	Southern	0.86	6.7	3.8	5.8	3.3
West Bengal	Eastern	1.04	1.0	1.0	1.0	1.0
All India			99.0	28.2	86.2	24.9

*Source: [35]

We now do a sensitivity analysis with regards to additionality determination. The case of lax additionality assumes that all wind power projects submitted are registered. The median case assumes that the rejection rate remains at the current level (2 out of 18 projects, i.e. 11%). The case of stringent additionality assumes that 50% of the projects are registered. In the lax additionality case, gross annual CER potential of wind power in India reaches 86 million. Similarly, based on the technical potential of wind power projects in India the CDM potential has been estimated as 25 million tonne. Among all the states in India, Gujarat has the largest CO_2 _emissions mitigation potential through wind power (19 million tonne) followed by Andhra Pradesh (15.6 million tonne), Madhya Pradesh (10.8 million tonne), Karnataka (12.5 million tonne), Rajasthan (8.9 million tonne), and so on (Table 6). The annual electricity generation by wind power projects based on the gross and technical potential is also given in Table 6. With 25% PLF of wind power projects the annual gross electricity generation potential has been estimated at 99 TWh whereas the annual technical electricity generation potential has been estimated at 28 TWh.

**Figure 5:**
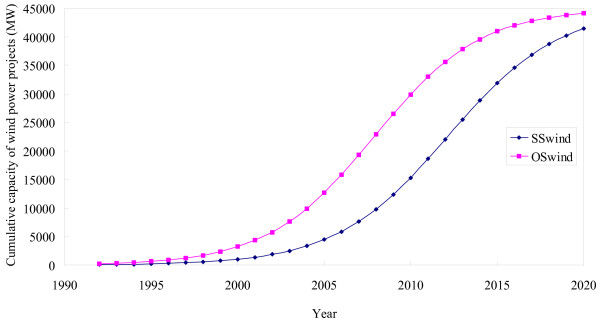
Realistic CDM potential for wind power until 2020.

Table 7 presents the projected values of the cumulative capacity of wind power and likely CER generation using the logistic model described in the Methods section while Figure 5 shows the development over time. It may be noted that with the current trend of dissemination of wind power projects in the country, around 22 GW capacity could be installed up to the end of first crediting period in the SS scenario whereas in the OS scenario 36 GW capacity could be installed. Up to the the year 2020, more than 44 GW capacity of the wind power projects are expected to be installed that would generate 87 million CERs.

**Table 7:** Projected values of the cumulative capacity of wind power and associated CER generation

Year	Projected values of the cumulative capacity (GW)		Projected values of the annual electricity generation (TWh)		Projected values of the annual CER generation (million CERs)	
	
	SS	OS	SS	OS	SS	OS
2008	10	23	21	50	18	43
2012	22	36	48	78	41	67
2016	35	42	76	92	65	79
2020	41	44	91	97	78	83

*Baseline 860 g CO_2_/kWh

## Discussion: How the CDM could be applied to the Diffusion of Wind Power Projects?

**Figure 6:**
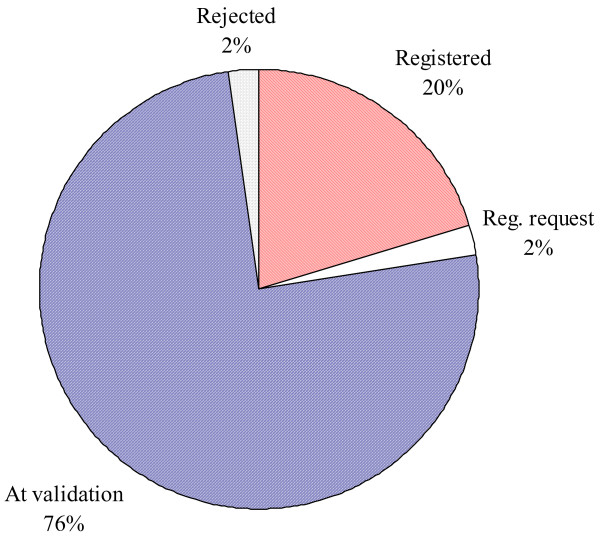
Status of the wind power projects from India till 20^th ^December 2006 (Source: [33]).

The CDM was slow to take off as after the Marrakech Accords of 2001 it took another three years to define the bulk of the rules. The CDM Executive Board (EB) which is the body defining the CDM rules surprised many observers by taking a rigorous stance on critical issues such as baseline and additionality determination (see below). Once the key rules were in place, a "gold rush" happened in 2005 and 2006. Over 1500 projects were submitted with an estimated CER volume of about 1.5 billion. However, the volume share of renewable energy projects has been less than expected due to the high attractiveness of projects reducing industrial gases and methane from waste. Out of the 1478 CDM projects submitted to the EB, 456 projects had been registered by the EB till 20^th ^December, 2006 [32,33]. 183 CDM projects related to wind energy of which 47 have been registered, 9 requested registration and 127 were at the validation stage [33]. Figure 6 presents the status of the wind power projects from India. Out of the 89 projects submitted to the UNFCCC, 18 projects had been registered and two projects had submitted the request for registration. 67 projects were at the validation stage whereas 2 projects had been rejected by the EB.

### Baseline

The quantification of GHG benefits of a CDM project is done by means of a "baseline". A baseline describes the (theoretical) emissions that would have occurred in case the CDM project was not implemented. The amounts of CERs that can be earned by the project are then calculated as the difference of baseline emissions and project emissions. The CO_2 _emissions mitigation benefits associated with a wind power project depend upon the amount of electricity saved. To estimate the CDM potential of wind power project in the country, the approved consolidated baseline methodology for grid-connected electricity generation from renewable sources ACM0002 (Version 06) has been used. For the small scale CDM (SSC) projects, the small scale methodology AMS-I.D. "Grid connected renewable electricity generation" in its version of 23^rd ^December 2006 [34] can be used which explicitly mentions wind power for electricity generation. In India, most of the wind power projects are grid connected and substitute grid electricity. Therefore, for such systems, the baseline is the kWh produced by the renewable generating unit multiplied by an emission coefficient (measured in g CO_2_eq./kWh) calculated in a transparent and conservative manner. This coefficient is 800 g CO_2_eq./kWh for a grid where all generators use exclusively fuel oil and/or diesel fuel, whereas it is the weighted average of the so-called operating margin (emission factor of all thermal power plants serving the grid) and build margin (emission factor of the most recently built plants that provide 20% of the grid's electricity). For wind power, the weight of the operating margin is 0.75 while the build margin is weighted at 0.25. Alternatively, project developers can use the weighted average emissions of the current generation mix but this will always be less than the emission factor derived previously and thus unattractive. For intermittent and non-dispatchable generation types such as wind and solar photovoltaic, ACM0002 allows to weigh the operating margin (OM) and build margin (BM) at 75% and 25%, respectively, however, in this study we have used combines margin by using equal weights for OM and BM as given in CEA document [35].

### Additionality

To maintain the environmental integrity of the Kyoto Protocol, CERs are given only for "additional" activities that would otherwise not be expected to occur [36]. Therefore, any CDM project requires careful analysis of additionality. This has probably been the most contentious point in the development of the CDM and also resulted in great confusion amongst project developers [37,38]. The Kyoto Protocol stops short of requiring project proponents to show strict financial additionality – that the CDM revenue makes an uneconomic project economic – and left scope for the CDM EB to refine the demonstration of additionality. The EB subsequently took a fairly strict interpretation of additionality and developed an additionality tool which formally is voluntary but which has become de facto mandatory as it was incorporated in most baseline methodologies. The additionality tool requires an investment analysis and/or a barrier analysis to determine whether the CDM project is the most attractive realistic alternative. This means that the project can be profitable and additional as long as developers can show that another project type was even more profitable.

It is estimated that wind power in many countries is already competitive with fossil fuel and nuclear power if social/environmental costs are considered [28]. However, in India, in terms of costs per kWh in grid-connected areas, costs of wind power are higher than electricity provided by a coal plant projects thus be additional at any rate. The unit cost of electricity generation is 0.05 €/kWh for coal and 0.06 €/kWh for fuel oil based system whereas in case of wind, the unit cost of electricity generation is 0.07 €/kWh in the best locations. The problem with this reasoning is that if wind projects are used to displace expensive grid electricity for industrial consumers (priced at 0.09 €/kWh [39]), they are invariably the most attractive alternative unless they are built in locations with low wind speed. The situation for wind projects that supply to the grid at the state-guaranteed feed-in tariff is less clear; the attractiveness depends on the level of the tariff.

As the investment test will not be passed by most wind projects (or only if they omit the tax incentives, as has been done by a project that achieved registration), project developers will use the barrier test. The barrier of higher capital cost compared to fossil fuel power plants is not really credible due to the abundance of capital for wind power in India and thus is mentioned only rarely. More credible barriers are low capacity utilization factor, and possible reduction in feed-in tariffs. The former depends on the siting of the project. The latter is very important as shown by the policy of Rajasthan (see Table 3) and other states. In 2001, Tamil Nadu Electricity Board (TNEB) changed its policy and froze the power purchase tariff for wind energy at Rs 2.70 per kWh with no escalation till 2006 and had informed that this power purchase tariff would be reviewed at 2006 and a new tariff would be fixed then. This was a major barrier for establishing new wind farms as other renewable energy plants continued to get a higher tariff. For instance, the power purchase tariff for electricity from an industrial waste/municipal waste based generation was Rs 3.49 for the year 2005 as against Rs 2.70 for wind energy. This policy encourages investors to invest in other renewable energy plants. Reduction in power purchase tariff was a major investment barrier. Moreover, feed-in-tariffs may be replaced by the Availability Based Tariff (ABT) in which the generators with firm delivery of power against commitment will start getting more prices for the generated power, whereas wind power producers cannot guarantee supply of electricity and will be thus receive lower rates. For the projects that substitute grid electricity at industrial tariffs, there is the risk that the wind power benefit will melt down as liberalization permits industrial electricity consumers to choose the supplier in a competitive environment. Some projects have also highlighted the technological risks associated with new types of wind turbines. Lack of familiarity and experience with such new technologies can lead to perceptions of greater technical risk than for conventional energy sources.

#### Doing the investment test – case study

A 125 MW wind project in Karnataka calculated an IRR of 7.3%. At that rate, the project would clearly be unattractive for an investor. However, the picture changes if one analyzes the project more closely. If one uses industry averages for the investment cost (Rs 5 crore per MW), the IRR is 11%. If one includes the accelerated depreciation of 80% in the first year and the 10 year income tax holiday, the IRR reaches 22% (personal communication by Mr. Sanjeev Chadha). It would be difficult to find serious alternatives that are more attractive. Nevertheless, the project was registered by the EB.

Table 5 presents the additionality arguments of Indian wind power projects. 14 projects out of 20 have carried out investment and barrier analysis for the justification of additionality whereas 6 projects carried out the barrier analysis only. An assessment of the PDD's indicates that the investment analysis is not convincing in most of the cases. Two wind projects from India were rejected due to lack of additionality. The rejection was mainly due to the following statement in the annual report of the company that had invested in the projects: "The project is extremely beneficial on a standalone basis and has a payback period of three years with an internal rate of return in excess of 28 per cent. In addition to hedging Bajaj Auto's power costs, this investment also provides sales tax incentives and an income tax shield" [40].

### Monitoring

For wind power plants, monitoring is easy – you just meter the electricity generated and sold to the grid.

## Conclusion

Our estimates indicate that, there is a vast theoretical potential of CO_2 _mitigation by the use of wind energy in India. On the basis of available literature, the gross potential of wind power is more than 45,000 MW. The annual CER potential of wind power in India could theoretically reach 86 million tonnes. Under more realistic assumptions about diffusion of wind power projects based on past experiences with the government-run programmes, annual CER volumes by 2012 could reach 41 to 67 million and 78 to 83 million by 2020. The projections based on the past diffusion trend indicate that in India, even with highly favorable assumptions, the dissemination of wind power projects is not likely to reach its maximum estimated potential in another 15 years. CDM could help to achieve the maximum utilization potential more rapidly as compared to the current diffusion trend if supportive policies are introduced.

## Methods

### CO_2 _emissions mitigation potential of a windmill

The power output of a windmill essentially depends on the site/location specific parameters (such as wind speed, air density, etc.) and design parameters (such as coefficient of performance of the wind rotor, swept area of the rotor, cut-in, cut-out and rated wind speed of the rotor, etc.) of the windmill. Therefore, the annual useful energy, AUE_wind_, delivered by a windmill can be estimated as [20]

AUEwind=8760γ∫vcivcoP(v)F(v)dv
 MathType@MTEF@5@5@+=feaafiart1ev1aaatCvAUfKttLearuWrP9MDH5MBPbIqV92AaeXatLxBI9gBaebbnrfifHhDYfgasaacH8akY=wiFfYdH8Gipec8Eeeu0xXdbba9frFj0=OqFfea0dXdd9vqai=hGuQ8kuc9pgc9s8qqaq=dirpe0xb9q8qiLsFr0=vr0=vr0dc8meaabaqaciaacaGaaeqabaqabeGadaaakeaacqWGbbqqcqWGvbqvcqWGfbqrdaWgaaWcbaGaem4DaCNaemyAaKMaemOBa4Maemizaqgabeaakiabg2da9iabbIda4iabbEda3iabbAda2iabbcdaWGGaciab=n7aNnaapedabaGaemiuaa1aaeWaaeaacqWG2bGDaiaawIcacaGLPaaacqWGgbGrdaqadaqaaiabdAha2bGaayjkaiaawMcaaiabdsgaKjabdAha2bWcbaGaemODay3aaSbaaWqaaiabdogaJjabdMgaPbqabaaaleaacqWG2bGDdaWgaaadbaGaem4yamMaem4Ba8gabeaaa0Gaey4kIipaaaa@520D@

where *γ *represents the windmill turbine mechanical availability factor accounting for downtime during maintenance etc., P(v) the power produced by the windmill at wind speed v (in m/s), F(v) the Weibull probability distribution function, v_ci _the cut-in wind speed and v_co _the cut-out wind speed of the windmill.

The power produced by the windmill at wind speed v may be expressed as [41]

P(v)=12CpρaAv3
 MathType@MTEF@5@5@+=feaafiart1ev1aaatCvAUfKttLearuWrP9MDH5MBPbIqV92AaeXatLxBI9gBaebbnrfifHhDYfgasaacH8akY=wiFfYdH8Gipec8Eeeu0xXdbba9frFj0=OqFfea0dXdd9vqai=hGuQ8kuc9pgc9s8qqaq=dirpe0xb9q8qiLsFr0=vr0=vr0dc8meaabaqaciaacaGaaeqabaqabeGadaaakeaacqWGqbaudaqadaqaaiabdAha2bGaayjkaiaawMcaaiabg2da9maalaaabaGaeGymaedabaGaeGOmaidaaiabdoeadnaaBaaaleaacqWGWbaCaeqaaGGacOGae8xWdi3aaSbaaSqaaiabdggaHbqabaGccqWGbbqqcqWG2bGDdaahaaWcbeqaaiabiodaZaaaaaa@3D62@

where C_p _represents the coefficient of performance of the wind rotor, *ρ*_a _the density of air, A the swept area of the rotor and v the wind speed.

The variation in wind speed at a location is often described by the Weibull probability distribution function F(v). The Weibull probability density function is given by the following expression [20,42]

F(v)=(kc)(vc)(k−1)e-(vc)k
 MathType@MTEF@5@5@+=feaafiart1ev1aaatCvAUfKttLearuWrP9MDH5MBPbIqV92AaeXatLxBI9gBaebbnrfifHhDYfgasaacH8akY=wiFfYdH8Gipec8Eeeu0xXdbba9frFj0=OqFfea0dXdd9vqai=hGuQ8kuc9pgc9s8qqaq=dirpe0xb9q8qiLsFr0=vr0=vr0dc8meaabaqaciaacaGaaeqabaqabeGadaaakeaacqWGgbGrdaqadaqaaiabdAha2bGaayjkaiaawMcaaiabg2da9maabmaabaWaaSaaaeaacqWGRbWAaeaacqWGJbWyaaaacaGLOaGaayzkaaWaaeWaaeaadaWcaaqaaiabdAha2bqaaiabdogaJbaaaiaawIcacaGLPaaadaahaaWcbeqaamaabmaabaGaem4AaSMaeyOeI0IaeGymaedacaGLOaGaayzkaaaaaOGaeeyzau2aaWbaaSqabeaacqqGTaqldaqadaqaamaalaaabaGaemODayhabaGaee4yamgaaaGaayjkaiaawMcaamaaCaaameqabaGaem4AaSgaaaaaaaa@47AC@

where k represents the shape parameter and c the scale parameter.

Substituting the values of P(v) and F(v) from Eqs. (2) and (3) into Eq. (1) the annual useful energy (in kWh) delivered by the windmill can be expressed as [43]

AUEwind=4.38(γCpρaAkck)∫vcivcov(k+2)e-(vc)kdv
 MathType@MTEF@5@5@+=feaafiart1ev1aaatCvAUfKttLearuWrP9MDH5MBPbIqV92AaeXatLxBI9gBaebbnrfifHhDYfgasaacH8akY=wiFfYdH8Gipec8Eeeu0xXdbba9frFj0=OqFfea0dXdd9vqai=hGuQ8kuc9pgc9s8qqaq=dirpe0xb9q8qiLsFr0=vr0=vr0dc8meaabaqaciaacaGaaeqabaqabeGadaaakeaacqWGbbqqcqWGvbqvcqWGfbqrdaWgaaWcbaGaem4DaCNaemyAaKMaemOBa4Maemizaqgabeaakiabg2da9iabbsda0iabb6caUiabbodaZiabbIda4maabmaabaWaaSaaaeaaiiGacqWFZoWzcqWGdbWqdaWgaaWcbaGaemiCaahabeaakiab=f8aYnaaBaaaleaacqWGHbqyaeqaaOGaemyqaeKaem4AaSgabaGaem4yam2aaWbaaSqabeaacqWGRbWAaaaaaaGccaGLOaGaayzkaaWaa8qmaeaacqWG2bGDdaahaaWcbeqaaiabbIcaOiabdUgaRjabgUcaRiabbkdaYiabbMcaPaaakiabdwgaLnaaCaaaleqabaGaeeyla0YaaeWaaeaadaWcaaqaaiabdAha2bqaaiabbogaJbaaaiaawIcacaGLPaaadaahaaadbeqaaiabdUgaRbaaaaGccqWGKbazcqWG2bGDaSqaaiabdAha2naaBaaameaacqWGJbWycqWGPbqAaeqaaaWcbaGaemODay3aaSbaaWqaaiabdogaJjabd+gaVbqabaaaniabgUIiYdaaaa@6570@

The annual gross CO_2 _emissions mitigation potential of a windmill essentially depends upon the annual electricity saved by the windmill and the CO_2 _emission factor of the electricity.

GCEwind=4.38(γCpρaAkck(1−l)) [∫vcivcov(k+2)e-(vc)kdv]CEFe
 MathType@MTEF@5@5@+=feaafiart1ev1aaatCvAUfKttLearuWrP9MDH5MBPbIqV92AaeXatLxBI9gBaebbnrfifHhDYfgasaacH8akY=wiFfYdH8Gipec8Eeeu0xXdbba9frFj0=OqFfea0dXdd9vqai=hGuQ8kuc9pgc9s8qqaq=dirpe0xb9q8qiLsFr0=vr0=vr0dc8meaabaqaciaacaGaaeqabaqabeGadaaakeaacqWGhbWrcqWGdbWqcqWGfbqrdaWgaaWcbaGaem4DaCNaemyAaKMaemOBa4Maemizaqgabeaakiabg2da9iabbsda0iabb6caUiabbodaZiabbIda4maabmaabaWaaSaaaeaaiiGacqWFZoWzcqWGdbWqdaWgaaWcbaGaemiCaahabeaakiab=f8aYnaaBaaaleaacqWGHbqyaeqaaOGaemyqaeKaem4AaSgabaGaem4yam2aaWbaaSqabeaacqWGRbWAaaGccqGGOaakcqaIXaqmcqGHsislcqWGSbaBcqGGPaqkaaaacaGLOaGaayzkaaGaeeiiaaYaamWaaeaadaWdXaqaaiabdAha2naaCaaaleqabaGaeeikaGIaem4AaSMaey4kaSIaeeOmaiJaeeykaKcaaOGaemyzau2aaWbaaSqabeaacqqGTaqldaqadaqaamaalaaabaGaemODayhabaGaee4yamgaaaGaayjkaiaawMcaamaaCaaameqabaGaem4AaSgaaaaakiabdsgaKjabdAha2bWcbaGaemODay3aaSbaaWqaaiabdogaJjabdMgaPbqabaaaleaacqWG2bGDdaWgaaadbaGaem4yamMaem4Ba8gabeaaa0Gaey4kIipaaOGaay5waiaaw2faaiabdoeadjabdweafjabdAeagnaaBaaaleaacqWGLbqzaeqaaaaa@71C1@

where l (in fraction) represents the electrical transmission and distribution losses of the grid and CEF_e _the baseline CO_2 _emission factor.

For a given capacity of a wind power project the CO_2 _emissions mitigation potential can be estimated as:

*GCE*_*wind *_= (*8760 PLF*_*wind *_*P*_*wind*_) *CEF*_*e*_

where P_wind _(in MW) represents the capacity of wind power project, PLF_wind _(in fraction) the plant load factor of the wind power project, CEF_e _the CO_2 _emission factor of electricity. The term inside the second bracket of the right hand side of Eq. (6) is the annual amount of electricity saved by the wind power project.

### Estimation of Diffusion of Wind Power Projects in India

The diffusion of a technology measured in terms of the cumulative number of adopters usually conforms to an exponential curve [44] as long as the new technologies manage to become competitive with incumbent technologies. Otherwise, the steep section of the curve would never be reached because technology use falls back to zero at the removal of subsidies [45]. The exponential growth pattern may be of three types – (i) simple exponential, (ii) modified exponential, and (iii) S-curve. Out of these three growth patterns, the simple exponential pattern is not applicable for the dissemination of renewable energy technologies, as it would imply infinite growth [46]. The modified exponential pattern (with a finite upper limit) is more reasonable but such a curve may not match the growth pattern in the initial stage of diffusion [47,48]. Empirical studies have shown that in a variety of situations the growth of a technology over time may conform to an S-shaped curve, which is a combination of simple and modified exponential curves [49,50]. The S-shaped curves are characterized by a slow initial growth, followed by rapid growth after a certain take-off point and then again a slow growth towards a finite upper limit to the dissemination [51]. However, a logistic model is used to estimate the theoretical cumulative capacity of wind power projects at different time periods.

As per the logistic model, the cumulative capacity, P(t), of the wind power projects disseminated up to a particular period (t^th ^year) can be expressed as [49]

P(t)=Pmax [e(a+bt)1+e(a+bt)]
 MathType@MTEF@5@5@+=feaafiart1ev1aaatCvAUfKttLearuWrP9MDH5MBPbIqV92AaeXatLxBI9gBaebbnrfifHhDYfgasaacH8akY=wiFfYdH8Gipec8Eeeu0xXdbba9frFj0=OqFfea0dXdd9vqai=hGuQ8kuc9pgc9s8qqaq=dirpe0xb9q8qiLsFr0=vr0=vr0dc8meaabaqaciaacaGaaeqabaqabeGadaaakeaacqWGqbaudaqadaqaaiabdsha0bGaayjkaiaawMcaaiabg2da9iabdcfaqnaaBaaaleaaieGacqWFTbqBcqWFHbqycqWF4baEaeqaaOGaeeiiaaYaamWaaeaadaWcaaqaaiabdwgaLnaaCaaaleqabaWaaeWaaeaacqWGHbqycqGHRaWkcqWGIbGycqWG0baDaiaawIcacaGLPaaaaaaakeaacqaIXaqmcqGHRaWkcqWGLbqzdaahaaWcbeqaamaabmaabaGaemyyaeMaey4kaSIaemOyaiMaemiDaqhacaGLOaGaayzkaaaaaaaaaOGaay5waiaaw2faaaaa@4BF1@

where P_max _represents the estimated maximum utilization potential of the renewable energy technology in the country. The regression coefficients a and b are estimated by a linear regression of the log-log form of equation as given below.

ln[(P(t)Pmax){1−(P(t)Pmax)}]=a+bt
 MathType@MTEF@5@5@+=feaafiart1ev1aaatCvAUfKttLearuWrP9MDH5MBPbIqV92AaeXatLxBI9gBaebbnrfifHhDYfgasaacH8akY=wiFfYdH8Gipec8Eeeu0xXdbba9frFj0=OqFfea0dXdd9vqai=hGuQ8kuc9pgc9s8qqaq=dirpe0xb9q8qiLsFr0=vr0=vr0dc8meaabaqaciaacaGaaeqabaqabeGadaaakeaaieGacqWFSbaBcqWFUbGBdaWadaqaamaaliaabaWaaeWaaeaadaWcaaqaaiabdcfaqnaabmaabaGaemiDaqhacaGLOaGaayzkaaaabaGaemiuaa1aaSbaaSqaaiab=1gaTjab=fgaHjab=Hha4bqabaaaaaGccaGLOaGaayzkaaaabaWaaiWaaeaacqaIXaqmcqGHsisldaqadaqaamaalaaabaGaemiuaa1aaeWaaeaacqWG0baDaiaawIcacaGLPaaaaeaacqWGqbaudaWgaaWcbaGae8xBa0Mae8xyaeMae8hEaGhabeaaaaaakiaawIcacaGLPaaaaiaawUhacaGL9baaaaaacaGLBbGaayzxaaGaeyypa0JaemyyaeMaey4kaSIaemOyaiMaemiDaqhaaa@51E4@

**Figure 7:**
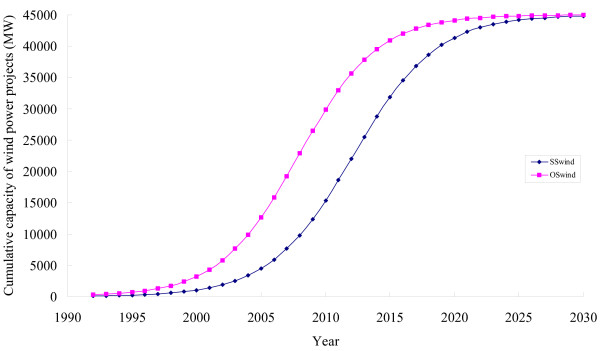
Time variation of cumulative capacity of wind power in India using logistic model.

Figure 7 represents the projected time variation of the cumulative capacity of wind power using the logistic model considered in the study. Two cases such as business as usual or standard scenario (SS) and optimistic scenario (OS) are presented. The values of the regression coefficients using a logistic model have been estimated by regression of the time series data for the installation of wind power (Figure 4) extracted from the annual reports of the MNES [25]. In the optimistic scenario it is assumed that, in the past, if the diffusion of wind power would have been driven by the market forces instead of subsidies then the cumulative capacity of installation of wind power would be three times more than the actual level [52,53]. Our results indicate that in India, even with highly favourable assumptions, the dissemination of wind power projects is not likely to reach its maximum estimated potential in another 15 years. But all these time periods are not relevant for the CDM whose current endpoint is 2012 and which may only be able to live longer if post-2012 negotiations retain an emission target based policy regime. However, CDM could be used as a tool to foster the dissemination of wind power projects in the country. It could accelerate the diffusion process.

## Abbreviations

ABT : Availability Based Tariff

BM : Build Margin

CDM : Clean Development Mechanism

CER : Certified Emissions Reduction

C-WET : Center for Wind Energy Technology

EB : Executive Board

GDP : Gross Domestic Product

GHG : Greenhouse Gas

IEA : International Energy Agency

IREDA : Indian Renewable Energy Development Agency

MNES : Ministry of Non-Conventional Energy Sources

NAL : National Aerospace Laboratory

OM : Operating Margin

PDD : Project Design Document

SERC : Structural Engineering Research Centre

SSC : Small Scale CDM

TNEB : Tamil Nadu Electricity Board

UNFCCC : United Nations Framework Convention on Climate Change

WEG : Wind Energy Generator

## Competing interests

Axel Michaelowa is working for CDM consultancy Perspectives, which has not been involved in wind power CDM projects in India

## Authors' contributions

Both authors have worked on all parts of the article, with Pallav Purohit focusing on the modeling and AM focusing on CDM issues.
